# Tick‐borne relapsing fever as a potential veterinary medical problem

**DOI:** 10.1002/vms3.108

**Published:** 2018-06-26

**Authors:** Nusirat Elelu

**Affiliations:** ^1^ Department of Veterinary Public Health and Preventive Medicine University of Ilorin Ilorin Kwara State Nigeria

**Keywords:** spirochaetes, *Borrelia*, *Ornithodoros*, *Argasid*, ticks

## Abstract

Tick‐borne relapsing fever (TBRF) caused by the bacteria *Borrelia*, is poorly documented in veterinary medicine. Given the widespread presence of the soft tick vectors – *Ornithodoros* and the recently discovered hard tick vectors, as well as their close association with animal hosts, it is highly likely that infection occurs, but is rarely reported to be of veterinary importance. Sporadic reports of canine infection, some being fatal through to probable cause of abortion in horses have been published. Some of these pathogens exist in regions where there are limited diagnostic facilities, hence, they are likely to be missed and their impact on productivity may be unquantified. Here we review available literatures on cases of TBRF in domestic and wild animals in order to show their potential veterinary medical impact. Future efforts using field and laboratory surveys are needed to determine pathogenesis, vector competence and distribution in animals, their impact on animal health and productivity as well as to prevent further spill to the human population, where it is already a public health problem in some parts of the world.

## Introduction

*Borrelia* species are tick‐borne, Gram‐negative, spiral‐shaped bacteria that causes several diseases across the world, grouped into Lyme borreliosis (LB) and tick‐borne relapsing fever (TBRF) (Socolovschi *et al*. 2009). Tick‐borne relapsing fever (TBRF) is a bacterial febrile illness caused by the spirochaete *Borrelia* (Vial *et al*. 2006). Tick‐borne relapsing fever is endemic and an important public health problem in some parts of the world. In Western Africa, the incidence of human tick‐borne relapsing fever (TBRF) is high, accounting for about 13% of febrile illnesses (Parola *et al*. 2011). In endemic regions of East Africa, TBRF borreliosis is one of the highest ranked causes of mortality among children (Talbert *et al*. 1998).

The organisms are transmitted either via saliva or in excreted coxal fluid of *Ornithodoros* soft ticks or some hard ticks including *Ixodes* and *Rhipicephalus* (*Boophilus*) species during feeding. These ticks are widely distributed in sub‐Saharan Africa, Asia, the Americas and some parts of Europe (Cutler 2006; Sato *et al*. 2014; Lopez *et al*. 2016). *Ornithodoro*s ticks often reside within cracks and crevices of animal dwellings, feeding indiscriminately on many different kinds of animals, including humans (Breitschwerdt *et al*. 1994). In North America, TBRF agents are principally: *Borrelia hermsii*,* Borrelia turicatae* and *Borrelia parkeri* transmitted by *Ornithodoro*s *hermsi, Ornithodoro*s *turicata* and *Ornithodoro*s *parkeri* respectively (Felsenfeld 1971; Dworkin *et al*. 2008; Lopez *et al*. 2016). In South America, *O. brasiliensis* is a potential vector of TBRF caused by *Borrelia brasiliensis* in the Southern Brazilian highlands (Martins *et al*. 2011). *Ornithodorus talaje* which are potential vectors *of Borrelia mazzottii* are also prevalent in South American countries including: Ecuador, Colombia, Venezuela, Argentina and Brazil (Guglielmone *et al*. 2006). *Borrelia persica* also referred to as the Persian relapsing fever and transmitted by *Ornithodorus tholozani* ticks, is the causative agent of TBRF in Central Asia and Middle Eastern countries including Iran, Israel, Egypt, Syria, Pakistan and Uzbekistan (Rebaudet & Parola 2006; Cutler *et al*. 2009; Elbir *et al*. 2014; Baneth *et al*. 2016). *Borrelia hispanica* has been reported in Spain, Portugal, Cyprus, Greece and North Africa. It has been isolated in the soft ticks, *Ornithodorus erraticus* (Rebaudet & Parola 2006). *Borrelia caucasica*, another agent of TBRF is present in Caucasus and Iraq and transmitted by *Ornithodorus asperus* (Rebaudet & Parola 2006). In East Africa, *Ornithodorus moubata* tick complex are known vector of *Borrelia duttonii* (Fukunaga *et al*. 2001; Mitani *et al*. 2004). Other *Borrelia* species documented in humans from different parts of Africa include: *B*. *crocidurae* common in Western Africa and transmitted by *Ornithodorus sonrai* with rodents and insectivores as reservoir host (Schwan *et al*. 2012). Others within Africa are *B. hispanica* transmitted by *O. erraticus* and small mammals as reservoir host (Trape *et al*. 2013). *Borrelia anserina* is the causative agent of avian spirochaetosis, with a worldwide distribution. It belongs to the relapsing fever (RF) group transmitted by *Argas* ticks and is not currently reported in man (Aslam *et al*. 2015).

A separate category of TBRF *Borrelia* are those transmitted by hard ticks such as *Ixodid*,* Rhipicephalus* and *Amblyomma* ticks, hereafter referred to as hard‐bodied tick‐borne relapsing fever (hTBRF) *Borreliae*. New species of *Borrelia* (*B. miyamotoi*) transmitted by hard‐bodied (*Ixodid*) tick species that transmit the Lyme borreliosis have been recently reported to cause relapsing fever in some humans in North America, Asian region of Russia, Europe and Japan (Platonov *et al*. 2011; Sato *et al*. 2014; Krause *et al*., 2015). *Borrelia theileri* is also a member of the hTBRF that causes spirochaetosis in cattle, sheep and goats (McCoy *et al*. 2014). In addition, there are other hard ticks such as *Rhipicephalus* and *Amblyomma* spp that have shown competence to serve as vectors of the relapsing fever *Borrelia*. For example, a study carried in Nigeria to determine pathogen prevalence in ticks reported 0.4% prevalence for *Borrelia* species in questing *Rhipicephalu*s *evertsi* ticks (the vector of bovine borreliosis). Although, sequence obtained from the 16S rRNA gene in that study from Nigeria for *Borrelia* species identification was unsuccessful, it showed 99% nucleotide homology to *B. burgdorferi* sensu lato (the Lyme disease pathogen), thus may possibly belong to a presently unclassified *Borrelia* species (Reye *et al*. 2012). The ability of hard ticks to transmit relapsing fever *Borrelia* has further expanded the potential geographical range of relapsing fever *Borrelia* group and stimulated much current research interest.

Many animals are potential reservoirs and final hosts for TBRF infection (Piccione *et al*. 2016). Natural vertebrate reservoirs of relapsing *Borrelia* include wild birds, rodents, chipmunks, squirrels, rabbits, owls and lizards (Breitschwerdt *et al*. 1994; Hamer *et al*. 2012). Both rodents and birds have been confirmed to act as reservoir hosts of *B. miyamotoi* (Wagemakers *et al*. 2017). A study carried out in Japan reported seabirds as potential reservoir hosts for relapsing fever because *Borrelia* spp closely related to *B. turicatae* was isolated from seabird tick vectors (Takano *et al*. 2009); and Carios ticks from bat in the USA (Schwan *et al*. 2009). Although TBRF is most likely under recognized and under diagnosed in veterinary medicine (Piccione *et al*. 2016), naturally occurring spirochaete infections have been detected in the blood of a variety of mammals, including squirrel monkeys, opossums, and armadillos, calves and horses (Dunn & Clark 1933; Lopez *et al*. 2016). The impact that these *Borrelia* spirochaetes have on the health of wild and domestic animals is largely understudied compared to the disease in humans (Schwan *et al*. 2005). This review compiles a comprehensive information of TBRF in various animal species around the world with the aim of identifying why it is an important differential in managing febrile illnesses in veterinary practice. In addition, we report the likelihood of its emergence in areas previously thought to be free of the disease especially with increasing close interaction between man and animals either from their domestication, encroachment on bushes, gaming and ranching.

## Tick‐borne relapsing fever in dogs and cats

Tick‐borne relapsing fever is a potentially fatal infection in pet animals such as dogs and cats (Baneth *et al*. 2016). Dogs are most likely to be fed upon by *Borrelia*‐infected *Ornithodoros* ticks when they sleep in tick‐infested cabins or while foraging in excavated, or underground burrows or caves (Kelly *et al*. 2014). Other likely sources of infection to dogs and cats are animal cages or cardboard boxes used to transport animals from suburban areas to cities for sale. These cages may harbour minute infected larval ticks and/or early stage nymphs that might be transferred with them thus serving as source of infections to pet animals and humans (Shirani *et al*. 2016). In 1939, Brumpt and Brumpt demonstrated that a 3‐week‐old dog was susceptible to infection with *B. turicatae* when fed upon by 10 relapsing fever‐infected *O. turicata* ticks (Brumpt & Brumpt 1939). More recently there has been confirmed reports of TBRF in canines from United States (Whitney *et al*. 2007; Kelly *et al*. 2014; Piccione *et al*. 2016). In addition, recent case reports from Israel and Iran in dogs with clinical manifestation resembling TBRF were confirmed using molecular methods to be infected with *B. persica* (Baneth *et al*. 2016; Shirani *et al*. 2016). More recently, there have also been confirmed reports of *Borrelia persica* as the cause of relapsing fever in cats (Schwarzer *et al*. 2015; Baneth *et al*. 2016). Infection of dogs and cats can, however, be complicated with co‐infection with other haemoparasites such as *Babesia* (Baneth *et al*. 2016). In the United State, *B. turicatae, B. hermsii* were the predominant cause of TBRF in dogs, these species of *Borrelia* are also the most important affecting humans in those areas (Kelly *et al*. 2014; Piccione *et al*. 2016). There are currently no reports of other species of relapsing fever *Borrelia* (*B*. *crocidurae, B. duttonii and B. hispanica*) causing pathogenic illnesses in dogs in Africa, where there are endemic foci of infection, and are pathogenic to human. This may warrant specific studies to determine the presence or absence of the disease in dogs because of the close interaction of dog with human and the reports of tick vectors in human dwellings where dogs are also resident.

Clinical presentation in dogs although nonspecific includes pyrexia, possible lethargy, anorexia, neurological signs (ataxia, tail tucking and cranial nerve deficits) (Piccione *et al*. 2016). Other common signs reported in dogs are fever, ambulation or postural defects (arched back, lameness), anorexia/weight loss and ocular lesions such as uveitis, corneal oedema (Breitschwerdt *et al*. 1994; Kelly *et al*. 2014). There is also report of persistent hind limb weakness and pain reported by Piccione *et al*. (2016), as well as lameness in the left rear leg with swollen stifle joint reported post treatment (Breitschwerdt *et al*. 1994). Haematological abnormalities in infected dogs include microcytic, normo‐chromic anaemia, slight poikilocytosis and severe thrombocytopenia (Breitschwerdt *et al*. 1994). The clinical signs in cats is similar to those reported in dogs and include mainly anorexia, lethargy, pale mucous membrane as a result of anaemia, some with icterus and haematology showing thrombocytopenia (Baneth *et al*. 2016).

## Tick‐borne relapsing fever in birds

*Borrelia anserina* is the causative agent of avian spirochaetosis. It is transmitted by the soft tick, *Argas* and manifests in birds as fever, ruffled feather, inappetence and greenish diarrhoea (Ataliba *et al*. 2007). An experimental study to determine the clinical manifestation of avian spirochaetosis due to *B. anserina* in Sudan, reported clinical signs including pyrexia, dullness, ruffled feather, weight loss, drop in egg production and paleness of comb and wattles (Nasri *et al*. 2010). Avian spirochaetosis presents a potential economic problem in places like Africa, where poultry are an important source of protein. A study carried out in Ethiopia, *Argas persicus* ticks were found to carry *B. anserina* in 7.5% of *A. persicus* tick pools (Cutler *et al*. 2012). In Nigeria, clinical cases of avian spirochaetosis have been reported (Sa'idu *et al*. 1995). Domestic chicken was shown to be a naturally suitable host for *Borrelia* with an infection rate of 11% reported in Tanzania, where *B. duttonii* is a common human infection (McCall *et al*. 2007). In North America, Schwan *et al*. (2007) successfully produced spirochaetaemia in chicken by inoculating *B. hermsii* subcutaneously (Schwan *et al*. 2007).

## Tick‐borne relapsing fever in cattle

*Borrelia theileri* transmitted by hard‐bodied ticks including *Rhipicephalus (Boophilus)* is the causative agent of bovine borreliosis. The disease has also been reported from cattle, sheep and horses from several countries in Africa, South America, Europe and Australia (Uilenberg *et al*. 1988; Bishop 1994; McCoy *et al*. 2014). It is often associated with babesiosis. The clinical signs observed include fever, haemoglobinuria, loss of appetite, diarrhoea, pale mucous membranes, enlarged superficial lymph nodes and rough hair coats (Sharma *et al*. 2000). However, the author in that study could not determine if the clinical was as a result of co‐infection of *Borrelia* and *Babesia*. Clinically infection is usually benign hence underreported especially if there is mixed infection.

## Tick‐borne relapsing fever in pigs

Although no clinical case has been reported so far, ticks that are vectors of *Borrelia* have been reported in pigpens ((McCall *et al*. 2007). Relapsing fever *Borrelia* that shared great homology with *B. duttonii* has been reported in domestic pigs with a prevalence rate of 8.9% in Tanzania (McCall *et al*. 2007). In Europe, *O. erraticus*, the soft tick vector of human *B. hispanica* is usually found in traditionally raised pig herd that are allowed to forage in non‐intensive systems (Palma *et al*. 2011). The role of pigs as potential reservoir hosts of the disease as well as clinical importance would warrant further studies.

## Tick‐borne relapsing fever in horses

There was a previous report of equine abortion due to TBRF spirochaete (*B. parkeri*) for which the mare might have been an incidental host for an infected tick (Walker *et al*. 2002). A past case–control study reported that TBRF *Borrelia* species in Africa is associated with high perinatal mortality of about 436/1000 births and abortion in pregnant humans which may be as a result of trans‐placental transmission of the pathogen (Jongen *et al*. 1997). Similar pathogenesis on the abortifacient ability of TBRF in humans might explain the equine abortion reported in that case report. Further epidemiological studies bearing in mind TBRF as a probable cause of equine abortion in endemic areas is suggested.

## Tick‐borne relapsing fever in wildlife

Relapsing fever *Borreliae* has been isolated from wild animals in areas where human outbreaks were reported and tick vectors present. Wild birds have been reported to maintain and move ticks and *Borrelia* pathogens by serving as pathogen reservoir hosts (Hamer *et al*. 2012; Wagemakers *et al*. 2017). *Borrelia miyamotoi* has been reported in wild turkey (*Meleagris gallopavo)* with a very high prevalence of 58% (Scott *et al*. 2010). In a study carried out in North America to characterize *Borrelia* species from argasid bat tick, *Carios kelleyi*, the isolates although genetically distinct but were closely related to *B. turicatae* and *B. parkeri* species of tick‐borne relapsing fever spirochaetes (Gill *et al*. 2008). Furthermore, *Borrelia hermsii* has also been detected in blood of mule deer during surveillance (Nieto *et al*. 2012); and was isolated from chipmunks (*Tamias umbrinus*), during human disease outbreak investigation of relapsing fever in United States (Trevejo *et al*. 1998; Christensen *et al*. 2015).

However, there was a recent discovery of a relapsing fever spirochaete causing fatal borreliosis in an infected juvenile female bat (*Pipistrellus* species) in the United Kingdom. A PCR‐based analysis targeting the *flaB* and *glpQ* gene fragments showed that the causative agent had close similarities with the relapsing fever *Borrelia* previously reported in Africa – a cluster containing *B. recurrentis, B. duttonii* and *B. crocidurae* (Evans *et al*. 2009). Also, suspected fatal borreliosis in Northern spotted owl has been reported in United States, the *Borelia* organisms isolated had 99.6% similarities with *B*. *hermsii* which is one of the agents of human borreliosis in United States (Fischer *et al*. 2009). The Northern spotted owls prey on small rodents (dusky‐footed and bushy‐tailed woodrats, northern flying squirrels and red tree voles) which are documented reservoir of relapsing fever *Borrelia* and might have been the source of infection (Thomas *et al*. 2002). Other sources of infection such as through consumption of brains of TBRF infected animals by wild animals have been documented in experimental models (Horrenberger 1955). A relapsing fever *Borrelia* was also isolated from the endangered African penguin, *Spheniscus demersus*. In that study, one bird was believed to have died of borreliosis based on gross, microscopic lesions and analysis of partial *flaB* gene sequences – which is specific to relapsing fever *Borrelia* (Yabsley *et al*. 2012).

Although most of the studies in wildlife documented their importance as reservoir hosts, the few fatal cases due to borreliosis such as reported in owl, penguin and bat shows that TBRF may be an important disease of wildlife species that might be missed or underreported. Efforts should be made to understand the disease epidemiology in these group of animals as they may be important reservoir hosts infecting human and domestic animal population.

## Diagnosis and treatment of tick‐borne relapsing fever

Diagnosis of TBRF in human is made by microscopic examination of Giemsa‐Wright stained blood smears during acute spirochaetaemia (Lopez *et al*. 2016), and has been widely carried out to detect the presence of the spirochaetes in animal blood samples (Baneth *et al*. 2016). Serological assays to detect *Borrelia* immunogenic protein A (*BipA*) and glycerophosphodiester phosphodiesterase (*GlpQ*) antigen have also been carried out and have shown usefulness in differentiating RF *Borrelia* and Lyme disease group, although caution should be taken in interpreting results as current or past infection (Schwan *et al*. 1996; Lopez *et al*. 2013). PCR‐based analysis of specific gene regions such as 16S rRNA, *flaB* and *glpQ* genes fragments has been effective in diagnoses and speciation of RF *Borrelia* (Schwan *et al*. 2005; Piccione *et al*. 2016). Although there are no specific diagnostic methods for TBRF in animals, these methods listed above have been employed in confirming suspected animal cases or during surveillance studies. Examination of Wright‐Giemsa stained blood smears has been shown to provide a rapid diagnosis of TBRF in dogs as only relapsing fever *Borrelia* causes spirochaetaemia that is detectable in blood smear (Breitschwerdt *et al*. 1994; Piccione *et al*. 2016). Other spirochaetes that are of veterinary importance include, *Leptospira* spp, *Brachyspira* spp. and *Borrelia burgdorferi* sensu lato (agent of Lyme disease), and they should be considered as differentials in animals presenting with similar clinical signs (Piccione *et al*. 2016). Molecular‐based methods have also been useful in confirming the species of relapsing fever *Borrelia* in past studies involving animals (Schwan *et al*. 2007; Evans *et al*. 2009; Takano *et al*. 2009).

Drugs used in treatment of human TBRF have shown usefulness in treating canine TBRF. There are very few information on specific treatment of TBRF in other species of animals. Doxycycline administered orally at the dose of 7.5 mg/kg for 6 weeks (Piccione *et al*. 2016) or doxycycline (200 mg q 12 h) and amoxicillin (400 mg q 12 h) for 14 days (Kelly *et al*. 2014) has been successfully used to treat TBRF in dogs. Tetracycline administered at the dose of 1 g every 8 h orally for 2 weeks, and triple antibiotic‐corticosteroid ophthalmic ointment every 6 h has also been successfully used in treating infected dogs (Breitschwerdt *et al*. 1994). Rapid recovery has been reported in cats treated with amoxicillin/clavulanic acid combination or with doxycycline and others treated with a combination of amoxicillin/clavulanic acid with long‐acting injectable tetracycline (Baneth *et al*. 2016). In case of avian spirochaetosis, clinically sick birds have been successfully treated with procaine penicillin, whereas those infected with *A. persicus* ticks were dusted with organophosphorus compound (Asuntol [coumaphos]) or carbamate (Kartzimet 20). Although Jarisch–Herxheimer reaction has been documented in humans following antibiotic treatment for RF borreliosis, so far there is no evidence of such reactions in animals (Baneth *et al*. 2016). However, this may be due to lack of adequate documentation of the disease in veterinary medicine, clinicians should therefore monitor closely animals being treated for RF borreliosis. It is also recommended that the poultry housing be treated for tick infestation (Sa'idu *et al*. 1995).

## Conclusions

**Figure 1:**
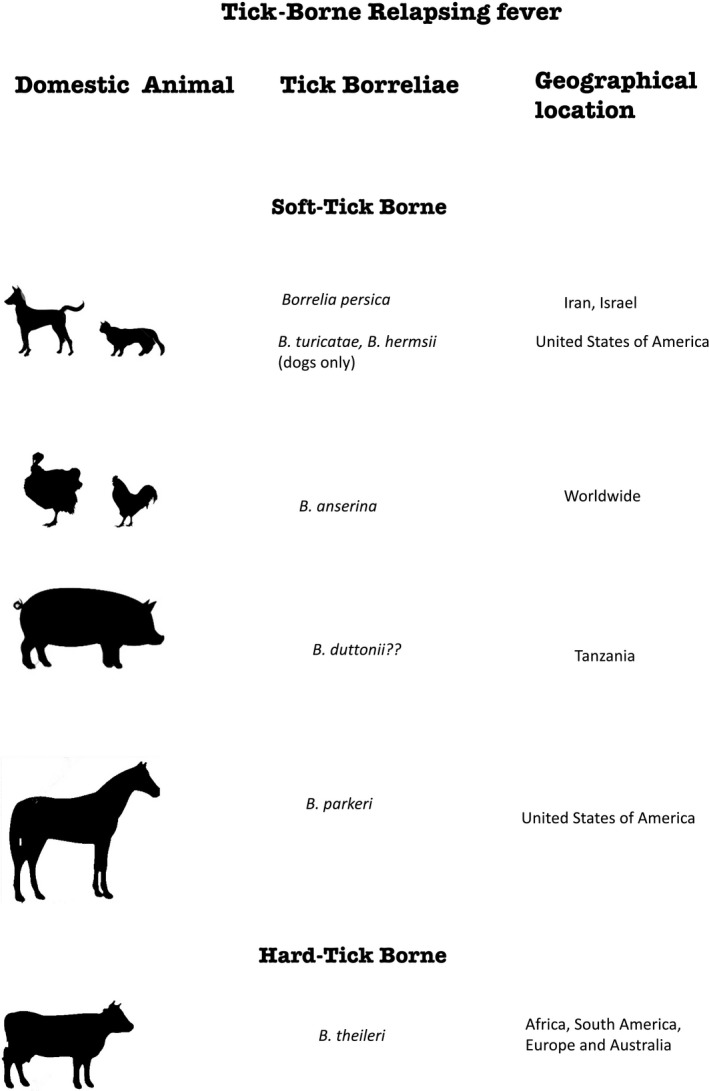
Tick‐borne relapsing fever borreliae of domestic animals and their geographical locations.

Although a number of studies have been carried out on hTBRF such as *B. theileri* in animals and world widely distributed *B. anserina*, majority of studies on other soft ticks TBRF in animals have are very few – summarized in Fig. 1. Some of these are case reports majorly in dogs. There are no reports of either suspect or confirmation of soft ticks TBRF in domestic animal population from most parts of the world. However, there are several reports of the TBRF in humans perhaps because it is a public health problem. With the existence of tick vectors in most places in Africa and across the world, TBRF is a likely animal health problem, should be investigated for, and included as differential in febrile case management that have accompanying similar clinical symptoms. In addition, further studies on the epidemiology of TBRF in domestic and indeed wildlife is suggested because of human co‐habitation with animals and their associated tick vectors could pose further public health risk.

## Conflict of interest

None to declare.

## Ethical statement

No ethical approval was required as this is a review article with no original research data.
